# Exploring the nano-wonders: unveiling the role of Nanoparticles in enhancing salinity and drought tolerance in plants

**DOI:** 10.3389/fpls.2023.1324176

**Published:** 2024-01-17

**Authors:** Abdul Rehman, Sana Khan, Fenlei Sun, Zhen Peng, Keyun Feng, Ning Wang, Yinhua Jia, Zhaoe Pan, Shoupu He, Lidong Wang, Abdul Qayyum, Xiongming Du, Hongge Li

**Affiliations:** ^1^ Zhengzhou Research Base, National Key Laboratory of Cotton Bio-breeding and Integrated Utilization, School of Agricultural Sciences, Zhengzhou University, Zhengzhou, China; ^2^ National Key Laboratory of Cotton Bio-breeding and Integrated Utilization, Institute of Cotton Research, Chinese Academy of Agricultural Sciences, Anyang, China; ^3^ Department of Plant Breeding and Genetics, University of Agriculture, Faisalabad, Pakistan; ^4^ Institute of Crop Sciences, Gansu Academy of Agricultural Sciences, Lanzhou, China; ^5^ National Supercomputer Center in Zhengzhou, Zhengzhou University, Zhengzhou, China; ^6^ Department of Plant Breeding and Genetics, Bahauddin Zakariya University, Multan, Pakistan

**Keywords:** drought, genetic engineering, nanoparticles, nanotoxicity, salinity

## Abstract

Plants experience diverse abiotic stresses, encompassing low or high temperature, drought, water logging and salinity. The challenge of maintaining worldwide crop cultivation and food sustenance becomes particularly serious due to drought and salinity stress. Sustainable agriculture has significant promise with the use of nano-biotechnology. Nanoparticles (NPs) have evolved into remarkable assets to improve agricultural productivity under the robust climate alteration and increasing drought and salinity stress severity. Drought and salinity stress adversely impact plant development, and physiological and metabolic pathways, leading to disturbances in cell membranes, antioxidant activities, photosynthetic system, and nutrient uptake. NPs protect the membrane and photosynthetic apparatus, enhance photosynthetic efficiency, optimize hormone and phenolic levels, boost nutrient intake and antioxidant activities, and regulate gene expression, thereby strengthening plant’s resilience to drought and salinity stress. In this paper, we explored the classification of NPs and their biological effects, nanoparticle absorption, plant toxicity, the relationship between NPs and genetic engineering, their molecular pathways, impact of NPs in salinity and drought stress tolerance because the effects of NPs vary with size, shape, structure, and concentration. We emphasized several areas of research that need to be addressed in future investigations. This comprehensive review will be a valuable resource for upcoming researchers who wish to embrace nanotechnology as an environmentally friendly approach for enhancing drought and salinity tolerance.

## 1 Introduction

In the current situation, population expansion has become a serious barrier to maintaining sustainable food production to meet the increasing population’s requirements (Calicioglu et al., 2019). The global population is expected to extend 9.6 billion individuals by the year 2050, necessitating a significant 70–100% increase in food output to meet the demands of this expanding population (Alabdallah and Hasan, 2021). Revolutionizing conventional agricultural practices is urgently necessary to attain the United Nations sustainable growth objective of “Zero Hunger” by 2030. These changes may be implemented through eco-friendly and sustainable approaches (Rajput et al., 2021b; Ranjan et al., 2021). Crop yields are significantly impacted by several issues, including increased abiotic stresses, declining fertile land, excessive fertilizer, pesticide usage, climate change and global warming (Hasan et al., 2021a; Hasan et al., 2021b). The drastic decrease in agricultural output is a serious hazard to world food security and a major problem. The required steps must be taken to lessen the damaging effects of abiotic stressors on crops if global food security is to be maintained. Therefore, implementing appropriate measures becomes imperative to address this issue effectively (Hasan et al., 2021a). Plants are immobile; hence they cannot relocate physically to escape the effects of environmental challenges, especially abiotic stress. Abiotic stresses like soil salinity and drought can cause considerable reduction in crop yield and quality (Haider et al., 2021; Yadav et al., 2021). Salt stress causes the cytosol to accumulate with Na^+^ and chloride Cl^-^ ions, severely damaging the cellular structure (Ranjan et al., 2021). The impacts of drought stress include the induction of stomatal closure, obstruction of photosynthesis, reduction of leaf area, inhibition of biomass and growth, reduction of water potential, elevation of osmolyte levels, and induction of reactive oxygen species (ROS) (Ibrahim et al., 2019). Hence, the modifications to plant metabolism under abiotic stress cause disturbances, resulting in the reorganization of the metabolic network to maintain crucial activities.

Over the last few decades, researchers have made great progress in creating a variety of stress management techniques. Among other strategies, nanotechnology is a highly effective strategy for significantly increasing crop yield (Shahid et al., 2020). However, most studies on NPs focused on examining their possible toxicity rather than their benefits (Ranjan et al., 2021). Nanotechnology has attracted much interest as an auspicious arena widely utilized in agriculture, food production, and medical (Alabdallah and Hasan, 2021). A tiny molecule cluster with an interfacial layer around it is called a nanoparticle with diameter 1 and 100 nanometers (Mohammadi et al., 2016). NPs have unique and uncommon features do not present in bulk materials because of their tiny size. Nanoparticle’s interesting scientific appeal arises from their capacity to link atomic or molecular structures to bulk materials (Khalid et al., 2022). NPs have been widely used in a variety of industries, including agricultural and allied ones as well as the chemical, optical, biomedical, pharmaceutical, culinary, and textile sectors (Verma et al., 2019). Due to their advantageous and safe uses, a number of NPs have recently attracted a lot of interest in the agriculture industry. These NPs comprise TiO_2_, Fe_3_O4, ZnO, SiO_2_, Cu-NPs, and Se-NPs (Alabdallah and Hasan, 2021; Hashem et al., 2021). There are precise methods to produce NPs, such as chemical, green, and physical synthesis (Akhtar et al., 2022). NPs can positively affect plant growth and development, although the precise impacts can vary depending on factors such as their time, origin, application, and size of crop administration (Rubilar et al., 2013). Current research exhibited that NPs protect plants by increasing the antioxidant metabolic activity to reduce oxidative damage (Ahmed et al., 2021). NPs regulate salt tolerance in diverse plant species by regulating hormone levels, antioxidant enzyme activities, ion balance, gene expression, and defense mechanisms (Zulfiqar and Ashraf, 2021). NPs can effects plant species and the environment in different ways like, NP shape, applications, and size, as well as environmental conditions, can influence their impact (Wahid et al., 2020). Nanopesticides and nano fertilizers are the two main use of nanotechnology in the agricultural industry.

**Figure 1:**
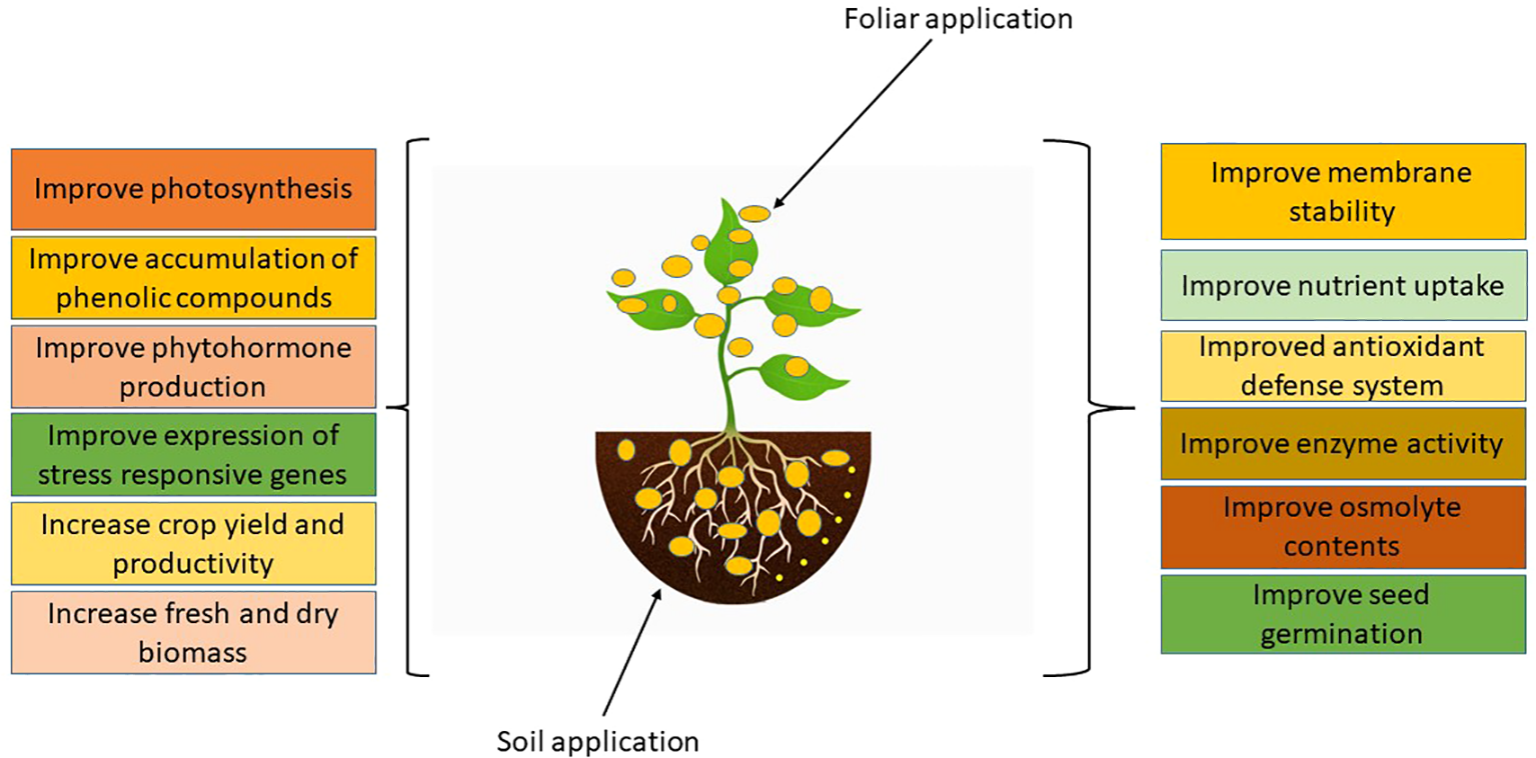
Impacts of NPs on plant growth and development.

Nanotechnology acts as a harbinger for a forthcoming industrial revolution. Agriculture’s productivity might be revolutionized by nanotechnology. According to a recent study, adding magnetic NPs through hydroponic system at seedling stage, significantly augmented the content of chlorophyll (a and b) and carotenoids in *Hordeum vulgare*. Additionally, the utilization of magnetic NPs favoured the genes related to photosystems (Tombuloglu et al., 2020). The utilization of NPs has been observed to mitigate the harmful effects of salinity and drought stress (Ali et al., 2021a; Singh et al., 2021). The effects of foliar spray of the NPs on plant growth and development are listed in **Figure 1**.

## 2 Classification of nanoparticle

**Figure 2:**
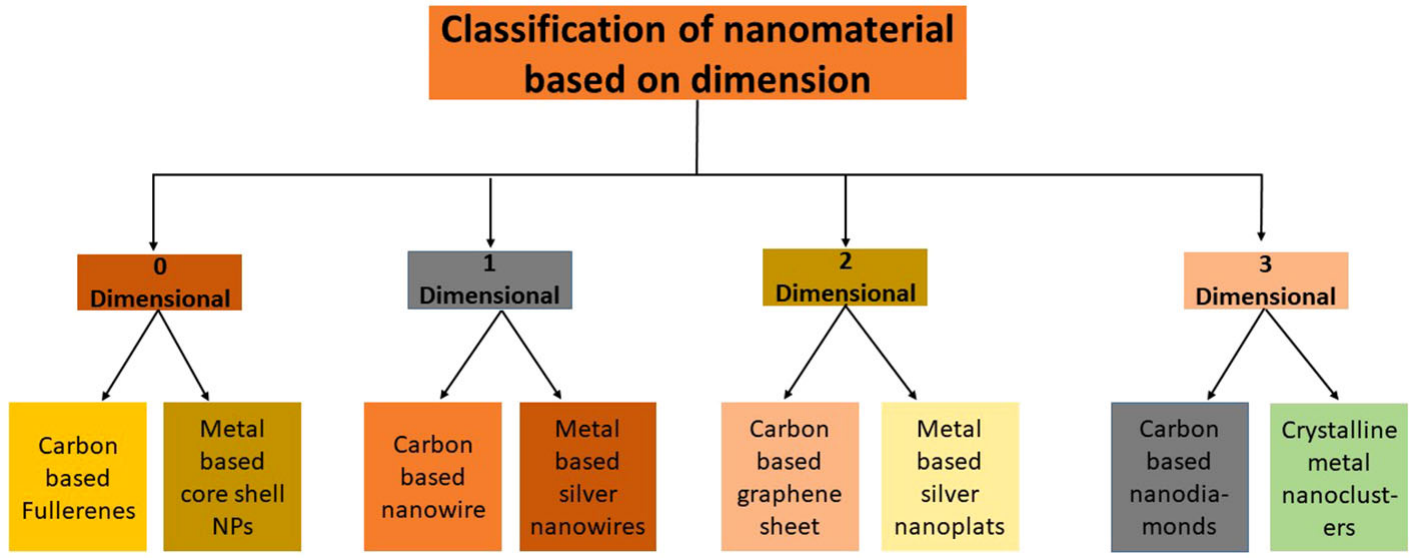
Classification of NPs based on dimension and composition.

The need for an appropriate categorization system for NPs eventually arose to advise scientists and engineers working on NPs studies and uses and encourage secure and more practical use of these resources. Studies have shown that the two most important factors in determining the classification of NPs are their dimensionality and composition. The need for an accurate categorization system to offer more convenience has grown during the last twenty years as the number of nanostructured materials has expanded (Prajitha et al., 2019). The “dimensionality” of the particles is the main criterion for categorizing NPs. According to Chung et al. (2013) NPs with zero dimension would effectively lack any observable dimension bigger than 100 nm in length. The production of zero-dimensional (0D) nanomaterials with precisely regulated dimensions has been made possible with the introduction of many physical and chemical manufacturing techniques (**Figure 2**).

Recently, numerous study groups have successfully improved various kinds of zero-dimensional (0D) nanomaterials, including but not limited to regular particle arrangements like quantum dots, varied particle arrangements, holy spheres, nano-lenses, and more (Kim et al., 2010). One dimension beyond the nanoscale distinguishes this class of nanomaterials, which we refer to as one-dimensional nanomaterials (1D). Nano horns, nanofibers, nanotubes nanowires, and nanorods are rare example. The two-dimensional (2-D) category of nanomaterials has two additional dimensions beyond the nanoscale. Nanolayers, nanofilms, and nanosheets are three prominent illustrations (Joudeh and Linke, 2022). Three-dimensional (3-D) materials, are not hindered by any dimension to the nanoscale. This category includes a variety of substances, including bulk powders, nanoparticle dispersions, arrays of nanowires, and nanotubes (Joudeh and Linke, 2022).

Next to dimensionality, the composition is also viewed appropriate criterion for nanoparticle categorization as it reliably reflects the chemical constitutes of the material. The categorization system includes NPs made entirely of carbon atoms as its initial category. This category contains substances like fullerenes, carbon nanotubes, graphene, and others. Carbon-based materials have several significant qualities, including exceptional strength and reactivity (Prajitha et al., 2019). The metal-based NPs, often known as metallic NPs, are categorized as the next group in this system. These NPs are made of metals with nanostructures, including titanium, gold, silver, and their equivalent oxides (Cataldo and Da Ros, 2008). This category also includes metal-decorated multi-walled nanotubes (MWNTs), metal nanoclusters, metal-filled single-walled nanotubes (SWNTs) and metallofullerenes. The optical characteristics of metallic NPs are vital in controlling their functions due to the peculiar surface plasmon resonance at visible wavelengths (Kumar et al., 2012). These polymeric NPs can contain the required therapeutic substance within their core due to their unique structural properties, or they can alternatively adsorb or be attached to their surface (Weir et al., 2012). The term “nanocomposites” refers to the next group in this categorization of NPs. Nanocomposites have been more popular over the past 25 years due to their wide variety of desired qualities that may be modified to meet particular needs (Kubiak, 2014). The distinctive physicochemical properties displayed by these materials result from the morphological and interstitial features of the component constituents.

## 3 Uptake, transformation and translocation of nanoparticles

Plants are the main producers in the environment, they are extremely important, and their interaction with NPs is a complicated process involving many variables. Plants are exposed to NPs found in soil and aerosols in their natural habitat (Azim et al., 2023). The uptake, translocation, and accumulation of NPs in plants are induced by various factors like the type, chemical composition, size, functionalization, root exudates, and microorganisms linked to the roots of the plant species (Etesami et al., 2021). The high mobility of NPs is governed by various factors, such as Brownian motion, Vander Waal forces, double-layer forces, and gravity. These elements are crucial for the adhesive properties of nanoparticles. NPs can enter into plants through air and soil when applied in liquid form (Azim et al., 2023). Aerosol NPs can reach plant aerial parts by various paths, including stomata, wounds, direct diffusion, or through aerial parts in contrast to soil NPs (Hussain et al., 2019). It is supposed that all the NPs behave differently in different plants and have different effects. Some researchers also explore that some NPs alter their shape and structure when exposed to plants like, silver, copper, and zinc oxide. It was observed that when ZnO NPs were applied on wheat they dissolve and release Zn ions in the root cells (Ali et al., 2021b). Likewise, CuO NPs transformed in to Cu(OH)_2_ and Cu_2_0 in the roots of the soybean plants (Kim et al., 2017). Moreover, Sliver NPs converted into silver chloride and silver sulfide in the leaf of the *Arabidopsis thaliana* (Thounaojam et al., 2021). Ensuring the validity and reliability of determining the shape, size, structure, and composition of NPs before and after exposure to plant tissue is a great challenge (Zhang et al., 2021). So, there is dire need to identify the exact, precise, pertinent, trustworthy, and appropriate method to characterize the NPs.

### 3.1 Nanoparticles transformations

NPs experience various modifications that are associated with their interactions, stability, and production in response to the environmental conditions. These modifications could be crucial in determining the properties and performance of NPs in different applications. The modifications carried out are of utmost importance to validate the safety, effectiveness, and overall influence of the NPs in plants.

#### 3.1.1 Stabilization and synthesis

NPs are synthesized through the alteration and transformation of precursor materials at the nanoscale level, resulting in the creation of distinct nanostructures. Diverse techniques are employed to synthesize NPs which include physical, biological, and chemical approaches. Various characterization methods play a crucial role in determining the physical properties of NPs, such as their size, shape, and surface properties. These factors have a significant impact on the performance and functionality of NPs, making their accurate determination essential for analyzing and optimizing their applications (Nel et al., 2006). NPs are often subject to surface modifications aimed at increasing their strength and enhancing their performance (Badawy et al., 2010).

#### 3.1.2 Aggregation and dispersion

NPs tend to either disperse or aggregate in the soil or water after their synthesis. The extent of dispersion or aggregation depends on multiple factors, such as the presence and concentration of organic substances, the pH level of the soil or water, and the concentration of various ions. Organic substances can either stabilize or destabilize the NPs, while the pH level and ion concentration can affect the electrostatic repulsion or attraction between the NPs. Understanding these factors is crucial for predicting and controlling the behavior of NPs in the environment (Azim et al., 2023). The stability, bioavailability, and movement of NPs are crucial factors that determine their interaction with roots. Prior to the interaction, these parameters play a pivotal role in determining the effectiveness and efficiency of the process (Baalousha et al., 2016).

#### 3.1.3 Surface coating and modification

NPs are often coated with a surface layer to improve their performance. These coatings can be modified using various factors such as ions, pH, and temperature. (Matzke et al., 2014). The process of surface coating plays a crucial role in the uptake of NPs, and also helps in controlling the germination and growth of plants (Biba et al., 2022). According to a recent study, it was found that the physiochemical properties of CRISPR Cas gene delivery are significantly influenced by the surface coating used. This highlights the importance of carefully selecting the coating material for effectiveness of NPs (Alallam et al., 2021).

#### 3.1.4 Chemical changes

The fate of NPs is significantly influenced by the oxidation and reduction reactions taking place in their surrounding environments. NPs composed of metals are particularly susceptible to oxidation, which can affect their interaction with plant tissues. Ultimately, the outcome of these chemical transformations plays a crucial role in determining the behavior and impact of NPs in different biological systems (Sotiriou and Pratsinis, 2010). The unique chemical properties of NPs can cause them to behave differently than bulk materials in terms of reactivity, conductivity, and strength. This is due to the high surface area to volume ratio of NPs, which can lead to increased surface reactivity and altered electronic properties (Verma et al., 2019; Staroń et al., 2020). As a result, it is important to consider the specific properties of NPs when designing and using them in various applications.

#### 3.1.5 Soil components and interaction

NPs display intricate interplay with the rhizosphere, encompassing a variety of factors such as microbes, minerals, organic matter, and other substances, which in turn, interact with the roots of plants (Ge et al., 2014). NPs are biologically inert and non-destructive, allowing them to persist in soil for extended periods. This can lead to alterations in soil microflora populations, soil fertility, and the metabolism and physiology of plant species (Pittol et al., 2017; Yang et al., 2017). The interactions demonstrate noteworthy impacts on the accessibility, transportation, and mobility of NPs in the soil system.

#### 3.1.6 Soil solution and transportation

The behavior of NPs in transportation is significantly impacted by various factors, such as dissolved ions, water content, and soil structure. Due to their small size, NPs display increased mobility in soil water. (Klaine et al., 2008). NPs can penetrate plant cells either through the transport system or endocytosis. Once inside, they accumulate in plant tissues and interfere with plant molecules, leading to disruptions in the plant’s physiological, morphological, and anatomical features and activities (Burman and Kumar, 2018; García-Gómez et al., 2018).

### 3.2 Mechanism of nanoparticles in drought and salinity tolerance

**Figure 3:**
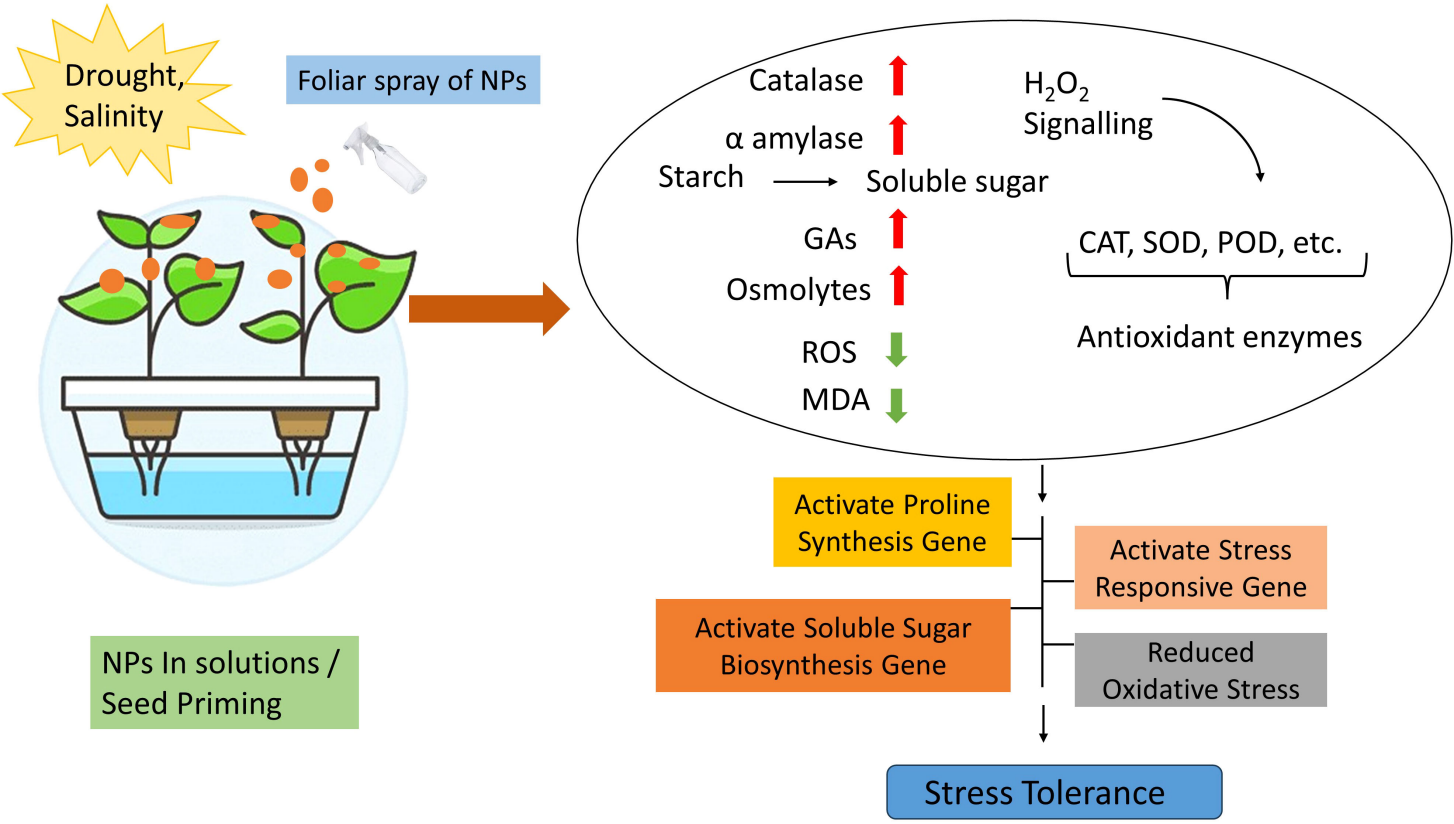
Molecular mechanism of NPs in drought and salinity stress tolerance.

NPs exhibited a significant role in increasing plant yield in drought and salinity. NPs demonstrated their potential to improve water loss by balancing water status ultimately abiotic stress tolerance (Rasheed et al., 2022). NPs also regulate stomatal conductance and transpiration rate through leaf anatomy and closing of stomata (Acosta-Motos et al., 2017). NPs demonstrated defensive impacts to protect photosynthetic machinery and increase photosynthesis in plants, also activate the antioxidant system to repair the damage caused by ROS in photosystems and chloroplasts. Moreover, NPs trigger electron transport chain and increase chlorophyll contents in plant cells (Forni et al., 2017; Manzoor et al., 2022). They also have influential effects on multiple physiological systems of plants including stress responsive mechanisms, hormone metabolism, biosynthesis of osmolytes, ethylene production, nitric oxide, ABA and calcium signaling. Additionally, regulate signal transduction pathways during salinity and drought stress and activate stress responsive genes, hence empowering the plant to cope and survive in stress conditions (Rasheed et al., 2022). Overwhelmingly, application of NPs has key role in plants systems to survive in drought and salinity and capable the plant to modulate its normal functionality, keep the plant and its environment healthy as well as maintain the plant yield (**Figure 3**). In-depth knowledge is still imperative to know the details functions of NPs to comprehend the stress tolerance in plants.

### 3.3 Seeds

Endocytosis offers a practical and effective method for internalizing extracellular substances like NPs. Endocytosis is a transmembrane process that occurs when the cell membrane is folded. The endocytosis of NPs can occur via clathrin-dependent or -independent routes. The number of parameters, such as size, charge, surface qualities, and other relevant parameters, substantially impact the uptake process of NPs (Azim et al., 2023). Metal-based NPs showed varied degrees of germination-unaffected seed penetration in wheat, maize, spinach, zucchini, rapeseed, and several desert plants (Chichiriccò and Poma, 2015). NPs may enter seeds and boost absorption, leading to better germination (Ahmadov et al., 2020). Multiwall carbon nanotubes (MWCNTs) were used to encourage the upregulation of tomato seedlings in terms of their seeds and root systems (Khodakovskaya et al., 2009). The beneficial influences of NPs on seed germination is also linked to their function in controlling aquaporins, tiny transmembrane water channels important for water permeability, seed germination, and plant development.

The research investigated the uptake and buildup of 8 nm ZnO nanoparticles in soybean seedlings across the 500-4000 mg range of exposure. At a dosage of 500 mg L-1, soybean seedlings showed a significantly higher uptake of Zn NPs in comparison to dosage 1000 mg L^-1^ or more. At lower dosage (500 mg L-1), NP aggregation seems to be less frequent. On the other hand, greater dosage (1000–4000 mg L-1) tend to lead to the formation of agglomerates (López-Moreno et al., 2010). The ability of gold NPs to translocate and concentrate within soybean plants after seed inoculation has been shown based on experimental findings (Maharramov et al., 2015). The thickness of seed coats makes it difficult for NPs to enter seeds when assessed to plant membranes and cell walls (Srinivasan and Saraswathi, 2010). Due to a mechanism known as enhanced water absorption, carbon nanotubes have demonstrated excellent penetration of the seed coat (Ganguly et al., 2014). It was discovered that nanotubes function as potential nano transporters, making it easier to carry DNA and tiny color molecules into whole plant cells (Savithramma et al., 2012).

### 3.4 Roots

According to studies, the rate at which NPs build up in plant roots may depend on environmental factors and NP characteristics (Ali et al., 2021a). It is believed that the small NPs can penetrate the roots of plants through capillary forces, osmotic pressure or by accurately passing through the epidermal cells of the roots (Ali et al., 2021a). Therefore, the semipermeable cell walls of the root epidermal cells have microscopic gaps that act as pores and prevent the passage of huge NPs. Furthermore, the cuticle performs as a key protective obstacle on leaves, successfully preventing NPs larger than 5 nm from penetrating the leaf. In general, determining the impacts of nanomaterials on the absorption, transport, and buildup of NPs within plants depends critically on their fundamental structure (Raliya et al., 2016). The term “apoplastic route” describes how holes in the root epidermal cell walls, generally between 5 and 20 nm in size, allow roots to absorb tiny NPs (Lin and Xing, 2007). In prior research, the content of Ag-NPs in both the shoot and roots of lettuce dramatically increased when silver sulphide (Ag_2_S) NPs were treated together with KCl and ammonium thiosulfate (Doolette et al., 2015). In another research, it was shown that organic matter in the soil reduced the amount of cerium dioxide (CeO_2_) NPs taken up by maize roots (Zhao et al., 2012). When applying NPs to soil or using them as a foliar application, findings have suggested that the mobility of NPs can be affected by bacteria associated with roots and leaves. Research has indicated that the use of NPs on soil or as a foliar application may affect the movement of NPs through the involvement of bacteria in the roots and leaves (Guo and Chi, 2014). The mucilage can also make the rhizosphere more acidic (Schaller et al., 2013), which encourages the disintegration of certain insoluble NPs (Schwab et al., 2016) and impacts on the extent to which plants absorb the NPs. Most research shows that the main restriction on entering NPs into plant cells is the size of the holes in the cell wall.

### 3.5 Leaves

The stomatal holes offer a different route for NPs to enter plants when examining foliar absorption (Larue et al., 2014). NPs can be transported to various plant parts, including the roots, through leaf translocation. Several plant species, including rapeseed, wheat, beans, corn, lettuce, and cucumber, show evidence of NPs internalization through their leaves (Chichiriccò and Poma, 2015). Leaves can internalize NPs of various sizes, varying from a few nanometers to several hundred nanometers, and are made of diverse materials, such as ceria, titania, FeO, ZnO, and Ag (Chichiriccò and Poma, 2015). Airborne NPs attach to plant leaf surfaces before being absorbed and entering epidermal cells. When NPs reach the epidermal cells, they have the potential to travel through apoplastic or symplastic pathways to numerous plant organs (Ahmadov et al., 2020). NPs are frequently sprayed onto leaf surfaces in agricultural applications, where they accumulate and are then taken up by plants via stomata or cuticle on the leaf surface. The primary constituents of the leaf epidermis’ waxy cuticle are wax, cutin, and pectin. The waxy cuticle of plant leaves is a crucial protective layer, preventing water loss when growing and acting as a key obstacle to inhibiting NPs from penetrating the leaves (Pérez-De-Luque, 2017). There are two different channels, lipophilic and hydrophilic channels, found on the waxy cuticle’s surface. Diffusion and penetration of lipophilic NPs into leaves are made possible by lipophilic channels on the cuticle surface (Bussieres, 2014). Confocal fluorescence microscopy with excellent temporal and spatial resolution was utilized by Hu et al. to demonstrate the ability of 2 nm carbon dots to penetrate cotton leaves via the cuticular route, showing their remarkable potential as a plant penetration-enhancing agent (Hu et al., 2020). Recent research has shown that various elements, including the properties of the NPs themselves, the plant species involved, and the surrounding environmental conditions, impact the absorption of NPs in plants. The immersion behavior of NPs in plant leaves can be affected by numerous properties such as chemical makeup, surface charge, particle size, and surface alteration (Wang et al., 2023). The particle magnitude of NPs has become a key component in investigating their absorption in the blade due to the size restriction limit of NPs in the blade absorption route (Li et al., 2020). NPs mostly travelled through the stomatal channel in the epidermis of wheat leaves before accumulating inside the chloroplasts.

## 4 Nanoparticles and their impact on drought and salinity stress

**Figure 4:**
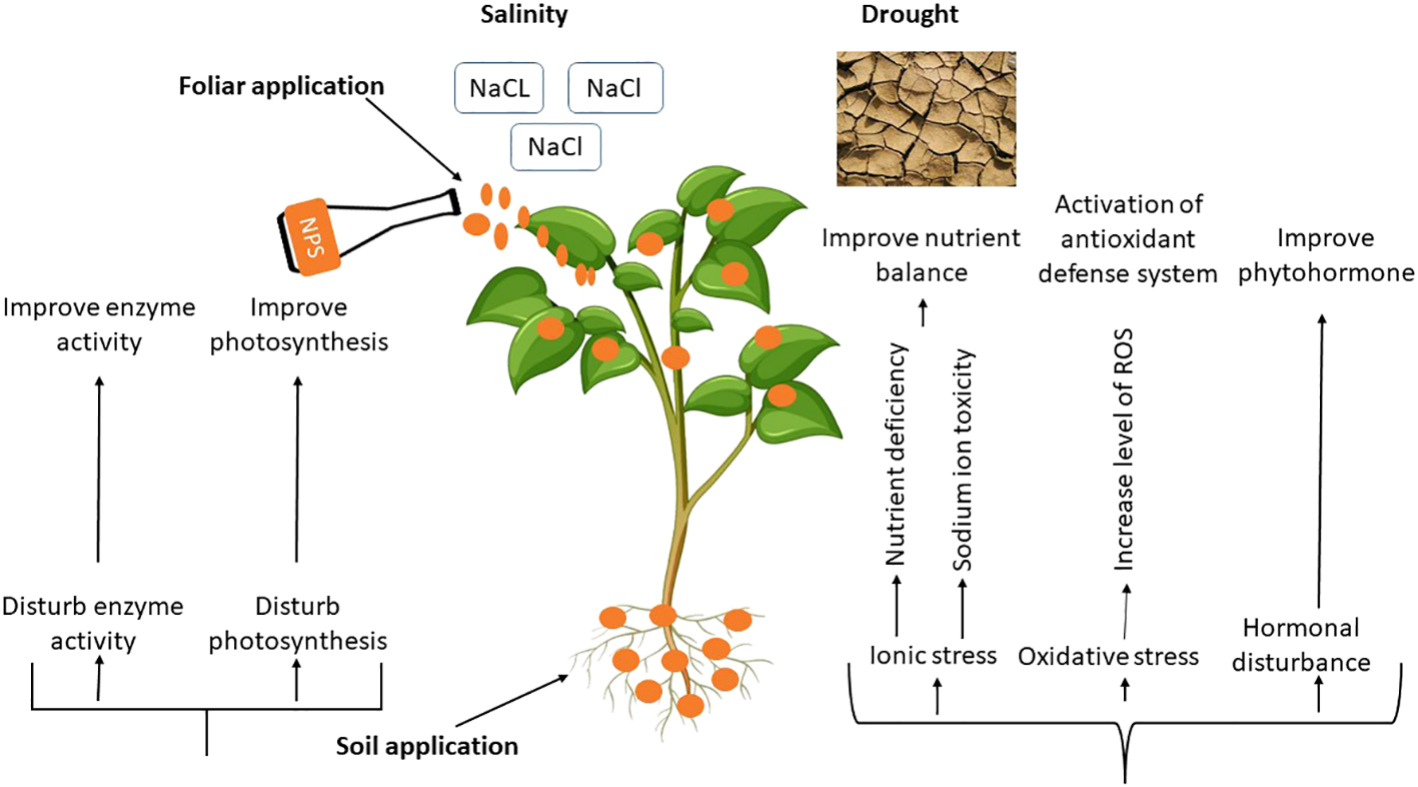
Physiological Mechanism of NPs induced drought and salinity tolerance in plants.

The entry points for NPs into the plant body are the roots and leaves, which can cause various biochemical, molecular, physiological and morphological changes in plants (Khan et al., 2019). The changes made have an important impact on the development of plants, which can differ based on the amount, method, and size of using the NPs. Plants’ physiological functions and overall health can be significantly impacted by the size, reactivity and chemical composition of NPs (Ahmed et al., 2021). According to the available research, various nanoparticle kinds may improve plant growth and development when subjected to salinity and drought stress (Ali et al., 2021a). Mechanism of NPs and their possible impacts under drought and salinity stress are depicted in **Figure 4** and **Table 1**.

**Table 1:** Impact of Nanoparticles in enhancing crop tolerance under drought and salinity stress.

NPS	Plant species	Stress	Effect	Reference
Zn	Soybean	Drought	Accelerated germination and decreased seed residual weight	(Sedghi et al., 2013)
Zn	Barley	Drought	Facilitated growth, increased production yield, added essential nutrients to edible grains, and improved nitrogen uptake.	(Dimkpa et al., 2019)
Zn	Maize	Drought	Increased melatonin synthesis and promoted antioxidant system function.	(Sun et al., 2020)
Zn	Sunflower and soybean	Salinity	Increased substomatal CO_2_ concentration, CO_2_ acclimatization ratio, chlorophyll content, as well as decreased leaf sodium (Na) and increased zinc (Zn) levels.	(Torabian et al., 2016)
Zn	*Lupinus termis*	Salinity	Induced plant development, repaired content of total phenols, organic solutes, and antioxidant enzymes, while lowering MDA levels.	(Abdel Latef et al., 2017)
Zn	Wheat	Salinity	Boost plant growth and development	(Fathi et al., 2017)
Zn	Tomato	Salinity	The concentrations of antioxidant enzymes were increased, leading to the stimulation of growth in roots and shoots, resulting in increased biomass and levels of photosynthetic pigments.	(Faizan et al., 2021)
Zn	*Trigonella foenum-graecum*	Salinity	Augmented proline and protein content, promoted antioxidant activity, and reduced MDA and H_2_O_2_ concentrations.	(Noohpisheh et al., 2021)
Zn	Mangifera indica L.	Salinity	Total sugars, Pro, and antioxidant enzyme levels have all increased.	(Elsheery et al., 2020)
Zn	*Ocimum basilicum*	Salinity	Improved growth characteristics are the result of increased pigment and Protein content.	(Kalteh et al., 2018)
Zn	*Lens culinaris* Medik.	Salinity	Reduced germination and seed activity, which reduced fresh weight (FW).	(Sabaghnia and Janmohammadi, 2015)
Zn	*Lycopersicum esculentum*	Salinity	Increased root weight and enhanced root development as a result of improved seed germination.	(Haghighi et al., 2012)
Zn	Fragaria sp.	Salinity	The RWC has increased as a result of the augmented chlorophyll and Pro concentrations.	(Avestan et al., 2019)
Zn	Hawthorn	Drought	Increased plant tolerance without having any discernible effects on the concentrations of carotenoid and chlorophyll.	(Ashkavand et al., 2015)
Ag	Lentil	Drought	Root length, FW, DW, and decreased germination ratio.	(Hojjat and Ganjali, 2016)
Ag	Tomato	Salinity	Improved Fresh and dry weight, root length and germination ratio of the seedlings.	(Almutairi, 2016b)
Ag	*Satureja hortensis* L.	Salinity	Improved growth metrics, such as shoot length, and higher germination percentages all contributed to a greater ability to withstand salt stress.	(Nejatzadeh, 2021)
Ag	Wheat	Salinity	Reduced the harmful consequences of salinity stress.	(Abou-Zeid and Ismail, 2018)
Ag	Wheat	Salinity	Seed germination efficacy is increased while oxidative stress is decreased because of the activation of antioxidant enzymes.	(Wahid et al., 2020)
Ag	Wheat	Salinity	Elevated concentrations of Pro and total sugar.	(Mohamed et al., 2017)
TiO_2_	Wheat	Drought	Enhanced growth, productivity, higher levels of transpiration rate, stomatal conductance, RWC, and chlorophyll and carotenoid concentration.	(Faraji and Sepehri, 2020)
TiO_2_	*Linum usitatissimum*	Drought	Decreased hydrogen, buildup and elevated chlorophyll, carotenoids, H_2_O_2_, and MDA concentrations.	(Aghdam et al., 2016)
TiO_2_	*Ocimum basilicum* L.	Drought	Reduced the negative impacts of drought and augmented RWC and biomass.	(Kiapour et al., 2015)
TiO_2_	*Zea mays* L.	Salinity	Improved seed germination efficacy reduced MDA, Pro and Na^+^ content while augmented K^+^ improved antioxidant and phenolic levels as well as FW, DW, and RWC.	(Shah et al., 2021)
TiO_2_	*Dracocephalum moldavica*	Salinity	Enhanced physiochemical characteristics by activating antioxidant processes.	(Gohari et al., 2020)
TiO_2_	Cotton	Drought	Augmented concentrations of total phenolic, total antioxidant capacity, and CAT, POD, and SOD activity.	(Shallan et al., 2016)
CeO_2_	Canola	Salinity	Efficiency Na+ was transported to shoots more effectively and Na+ buildup was reduced by lowering root apoplastic barriers. This led to a rise in plant biomass and an improvement in the photosynthetic system’s effectiveness.	(Rossi et al., 2016)
Chitosan	Wheat	Drought	RWC, High leaf area, photosynthetic rate,chlorophyll content, CAT and SOD activities, biomass and crop yield.	(Behboudi et al., 2019)
Chitosan	Barley	Drought	Improved grain weight, protein, RWC, pro content, CAT, and SOD activities.	(Behboudi et al., 2018)
Mn_3_O_4_	Cucumber	Salinity	Salinity increased the amount of photosynthetic pigment, net photosynthesis, and biomass, which altered the metabolomes.	(Lu et al., 2020)
Fe	Sorghum	Salinity	Improved the rate of photosynthetic activity, the chlorophyll index, the PSII effectiveness, RWC, and reduced lipid peroxidation.	(Maswada et al., 2018)
Fe	Moldavian balm	Salinity	Augmented leaf length, FW, DW, and leaf area per shoot and per root.	(Moradbeygi et al., 2020)
Fe	*Arabidopsis thaliana*	Drought	Boost biomass, photosynthetic pigments, and internal CO2 concentrations as a result of stimulated H+-ATPase activity controlling stomatal opening and closing.	(Kim et al., 2015)
Fe	*Triticum aestivum*	Drought	Better growth indices and higher photosynthetic activities as a result of increased absorption.	(Adrees et al., 2020)
Se	Wheat	Drought	The plant had better development and an increase in biomass by maintaining the chlorophyll and carotenoid concentrations and the leaf hydration status.	(El-Saadony et al., 2021)
CeO_2_	Glycine max	Salinity	The modulation of photosynthesis, water usage effectiveness, and Rubisco carboxylase in Glycine max boosted plant development and augmented the rate of photosynthesis at a concentration of 100 mg/kg of CeO2 NPs.	(Cao et al., 2017)
Cu	Maize	Drought	Enhanced the protective mechanism of maize during drought conditions.	(Van Nguyen et al., 2021)

### 4.1 Improve nutrient uptake

Plant development is negatively impacted by nutritional deficiencies and imbalances caused by drought stress, which disturbs the balance of nutrients inside plants (Umair Hassan et al., 2020). The nutrient absorption, transport, and distribution processes across different plant tissues are significantly improved by NPs, which also play a critical role in maintaining nutritional balance in plants (Kopittke et al., 2019). Plants with less water had lower N, K, Mn, and Zn levels due to reduced transpiration flux, nutrient uptake and compromised membrane stability (Semida et al., 2021). The absorption of N, phosphorous (P), K, and zinc was greatly enhanced by the treatment of NPs (ZnO) via foliar application and soil, and the negative impacts of drought stress were lessened (Akhtar et al., 2022). Maize plants that were cultivated under drought stress showed significant improvement in various parameters like photosynthesis, relative water content, antioxidant activity, and nutrient intake when they were co-treated with Si-NPs and plant growth-promoting rhizobacteria (Hafez et al., 2021). Adding zinc NPs (Zn-NPs) to wheat and sorghum plants increased productivity and improved nutrient absorption significantly (Dimkpa et al., 2019). NPs considerably increased nutrient intake, nitrate reductase activity, and nitrogen assimilation, enhancing protein and amino acid synthesis (Yuan et al., 2013). Using NPs boosts the appropriation of nutrients towards plant roots and improves plant nutrient absorption (Jaberzadeh et al., 2013).

Salinity stress is recognized to cause an imbalance in nutrient levels, resulting in excessive nutritional deficiencies in plants (Etesami and Maheshwari, 2018). It has been shown that Nano SiO2 increased the K^+^ amount in the leaves, which in turn improved the development of soybean seedlings under salt stress conditions (Farhangi-Abriz and Torabian, 2018). The exogenous spray of Cu-NPs was stated to reduce salinity stress in different research on tomato plants by promoting growth and preserving a balanced Na^+^/K^+^ ratio (Pérez-De-Luque, 2017). Abdoli et al. (2020) applying Fe_2_O_3_ NPs helped *Trachyspermum ammi* plants reduce salinity stress by rising the K^+^/Na^+^ ratio and Fe content. The research showed by Liu et al. (2021) augmentation of the K^+^-to-systolic sodium ratio in cotton plants was identified as one of the key processes underlying the improved plant growth observed upon the treatment of Ce NPs under salt stress. The results of this experiment show that the treatment of these NPs enhanced the amount of potassium in cells, which is a method to improve plant stress resistance. After using NPs, maintaining a balanced nutritional profile inside the plants is crucial to maximizing plant development under salt stress. For instance, research suggests that using Zn NPs can improve nutritional uptake of Zn. The transportation and intake of various nutrients, such as phosphorus, can be hampered when Zn absorption rises, causing an imbalance in the Zn-to-P ratio (Hussein and Abou-Baker, 2018).

### 4.2 Improve membrane stability

Plant development is significantly reduced by drought stress, which has negative effects on cellular membranes and the interactions between plants and water (Umair Hassan et al., 2020). Drought stress results in the production of ROS, impacts lipid peroxidation damages cell membranes, and leads to a build-up of high levels of MDA (malondialdehyde) (Das and Roychoudhury, 2014). The foliar spray treatment of hydrogen peroxide (H_2_O_2_), ZnO NPs, and MDA accumulation was significantly decreased, and membrane stability was maintained, preventing the deficiency of vital osmolytes (El-Zohri et al., 2021). However, when NPs (ZnO) were added exogenously, they were critical in preserving membrane integrity and cell water status during drought stress, which led to better PS-II efficiency and metabolic activities (Semida et al., 2021). The treatment of NPs preserved cell integrity and membrane stability, which boosted water absorption by improving the anatomy of the plants (Hafez et al., 2020). The relative electrolyte leakages of the maize membrane were dramatically decreased by priming with TiO_2_ NPs. This demonstrated that the protective effect of TiO_2_ against membrane deterioration caused by salt stress (Shah et al., 2021). The foliar treatment with CsNPs and modified CsBMs improved defense-related genes, JA signaling, anthocyanins, membrane stability, and diterpene glycoside synthesis under salt stress (Balusamy et al., 2022). Hence, NPs improved membrane stability, chloroplast formation, and sugar accumulation, all of which contributed to the improvement overall.

### 4.3 Improve enzyme activities

It was discovered that the use of 50 mg/L Cu NPs caused a substantial elevation of the gene expression level of SOD rose six-fold (Mosa et al., 2018). In contrast, ZnO NPs reduced the SOD activity of *Cicer arietinum* (Burman et al., 2013). The increases in the levels of numerous antioxidant enzymes GR, (Catalase), APX, SOD, CAT and GPX within plants suggest that applying fullerene NPs (FNPs) to the leaves may have positive effects in lowering the oxidative stress induced by drought stress (Liu et al., 2016). According to Taran et al. (2017), ZnO NPs increased wheat antioxidant enzyme (SOD and CAT) activity, which improved drought resistance.

Additionally, it was showed that bulk zinc oxide NPs caused higher stress in horticultural crops (Rajput et al., 2021a). According to a different study, applying selenium NPs and copper NPs to *S. lycopersicum* demonstrated increased crop production, higher levels of vitamin C, glutathione, chlorophyll, and increased activity of antioxidant enzymes such GPX, SOD and PAL (Hernández-Hernández et al., 2019). The research revealed that the action of both POX and SOD enzymes were greater in plants treated with the substance’s nanoform than those preserved with the bulk form, except for CAT activity (Ghorbanpour et al., 2015).

Using nano-sized SiO2 and TiO_2_ together has triggered a similar protective mechanism that improves fertilizer and water consumption and increases nitrate reductase activity in soybean (Changmei et al., 2002). Numerous nitrogen metabolism-related enzymes, including glutamate dehydrogenase, NiR, GS, and GPT, are controlled by titanium dioxide NPs (TiO_2_ NPs) in different plant species. This control allows the transformation of inorganic nitrogen into organic nitrogen, which is then converted into proteins, chlorophyll, and amino acids, as well as the absorption of nitrate and increased plant biomass (Mishra et al., 2014). According to Shah et al. (2021), TiO_2_ NPs defend the chloroplast from intense light by boosting the activity of antioxidant enzymes such CAT, POD, and SOD.

### 4.4 Improve phytohormone production

It is well known that phytohormones play crucial roles in assisting plants in becoming acclimated to various environments through various processes. Studies frequently show that phytohormones improve a plant’s capacity to endure salt stress (Fahad et al., 2015). Some studies have suggested that plant’s improved stress resistance is responsible for the alteration in plant hormonal balance that NPs induce (Paramo et al., 2020). In particular research, the presence of silver NPs caused substantial changes in the concentrations of ethylene, gibberellin and abscisic acid (ABA) in rice plants (Manickavasagam et al., 2019). The treatment of Ag-NPs used as priming agent at 1 mg L^-1^ on wheat plants improved the amounts of α-napthaleneacetic acid (NAA), 6-benzyl aminopurine (BAP), and indole-3-butyric acid, while concurrently reducing the content of ABA. These modifications were considered a crucial mechanism by which silver NPs boost plant development in challenging environments (Abou-Zeid and Ismail, 2018). The sixth hormone in plants, brassinolide (BR), is important for increasing cell elongation and division and boosting resistance to salt, drought, and heat stressors (Hou et al., 2018).

NPs reduce oxidative stress by controlling the buildup of osmolytes and hormones by boosting the antioxidant machinery (Silva et al., 2022). The treatment of NPs increases plant performance under drought stress circumstances by up-regulating the production of proline and sugars, which in turn helps preserve the integrity of cellular membranes, proteins, and enzymes (Gohari et al., 2020). Likewise, the utilization of NPs, specifically TiO2, substantially increases the buildup of phenolic substances, proline, glycine betaine, soluble sugars, and total proteins and improves plant development under drought stress (Mustafa et al., 2021). According to research by (Khan et al., 2019), mesoporous silica NPs (MSNs) that responded to glutathione were used to successfully distribute abscisic acid (ABA) to plants. The AtGALK2 was upregulated due to the limited discharge of ABA from MSNs, which eventually improved *Arabidopsis thaliana*’s ability to withstand drought. The treatment of NPs promotes the development of plants under drought stress by improving the production of indole acetic acid and gibberellins (GA) (Li et al., 2021). Using Fe-NPs and salicylic acid, a crucial plant growth hormone, dramatically increased the yield of strawberries (Mozafari et al., 2018). Sun et al. (2020) claim that the increased melatonin production caused by NPs was responsible for the induction of greater drought tolerance, suggesting that NPs may be able to cope drought stress via modulating endogenous hormones. In conclusion, maintaining hormonal balance with the treatment of NPs can increase plant’s ability to withstand drought and salinity.

### 4.5 Improve the accumulation of phenolics compounds

NPs can dramatically increase the accumulation of phenolic compounds in plants, whereas drought stress significantly reduces this accumulation. ZnO-NPs applied externally at 25 and 50 mg/L doses raised phenolic compound levels under drought stress, resulting in higher antioxidant activity and reduced MDA and H_2_O_2_ buildup (El-Zohri et al., 2021). The ZnO-NPs were investigated for their impact on the phenolic content of *Stevia rebaudiana*. The study exposed that the treatment of ZnO-NPs at concentrations of both 100 and 1000 mg/L resulted in significant reduction of the phenolic concentration (Javed et al., 2017). Additionally, plants treated with NPs have been shown to have a significant increase in non-enzymatic activities and total phenolic compounds, which lower lipid peroxidation and lessen oxidative damage (Ghani et al., 2022). The amounts of anthocyanin, phenolic, and antioxidant activity were increased after the treatment of Si- and Se-NPs (Zahedi et al., 2021). The growth and development of maize depend on the control of proteins and phenolic chemicals regulated by SiO_2_ NPs (Suriyaprabha et al., 2012).

The synthesis of phenolic compounds is known to rise in response to diverse abiotic stimuli because of its critical function in scavenging free radicals and antioxidants (Król et al., 2014). External application of TiO_2_ considerably increased the total phenolic content in *Vigna radiata*, which is consistent with earlier results that TiO_2_ seed priming efficiently controls phenolic compound synthesis in the maize hybrid under salt stress (Qi et al., 2013). The study carried out by Moradbeygi et al. (2020) aimed to investigate the effects of Fe NPs on *Dracocephalum moldavica* L. in salt stress circumstances. According to the experimental results, NPs enhanced plant development under salt stress by raising the abundance of flavonoid and phenolic chemicals, particularly in the roots, and lessening the activity of antioxidant enzymes. Additionally, compared to the control group, Cu NPs showed a considerable increase in glutathione, polyphenols, and vitamin C content under salt stress (Rajput et al., 2021a). The levels of total phenols, Osmo protectants, tannin, anthocyanins and flavonoids under salinity stress were also enhanced by the treatment of sulfur NPs to lettuce (Najafi et al., 2020). Additionally, AgNPs augmented the amounts of flavonoids and phenolic compounds while suppressing the leaf Na^+^/K^+^ ratio (Khan et al., 2020). For instance, foliar-applied Cu NPs in tomato plants reduced salinity stress by promoting growth and controlling the Na^+^/K^+^ ratio and growth (Pérez-Labrada et al., 2019). In contrast, CuNPs significantly amplified the amounts of phenols by 16%, vitamin C by 80%, glutathione by 81%, and phenols by 7.8%. Additionally, *Helianthus annuus* plants grown in a salty environment profited from the foliar treatment of Fe NPs because it improved the activities of polyphenol oxidase, CAT, and POD (Torabian et al., 2018).

### 4.6 Shield photosynthetic system and expand photosynthesis

Drought stress reduce the amount of chlorophyll produced, the effectiveness of PS-II, and the overall efficiency of photosynthesis in plants is adversely impacted (Semida et al., 2021). ZnO NPs enhanced chlorophyll synthesis, the activity of chlorophyll-synthesizing enzymes like chlorophyllase and fluorescence. As a result, photosynthetic efficiency increased under drought stress conditions (Abd El-Mageed et al., 2021). The external treatment of NPs aids in the stabilization of the ultrastructure of mitochondria and chloroplasts, which aids plants to sustain photosynthetic efficiency in drought stress (Rahmatpour et al., 2018). Titanium dioxide NPs are used to speed up the process of light-induced water hydrolysis, which releases oxygen, electrons, and protons (Silva et al., 2022). The efficiency of plants’ photosynthetic process subsequently improves significantly due to the faster entrance of protons and electrons into ETC (electron transport chain) (Alabdallah and Hasan, 2021). Titanium dioxide NPs foliar application increases the levels of photosynthetic pigments and boosts the gas exchange properties of plants by enhancing the activity of enzymes responsible for CO_2_ fixation and chlorophyll synthesis (Faraji and Sepehri, 2020). NPs further enhance the absorption of light within the chloroplasts, resulting in improved electron transport, enhanced efficiency of PS-II, increased O_2_ progression, and more efficient photo-phosphorylation, improved photosynthetic efficacy in plants under shortage of water (Shafea et al., 2017).

Photosynthesis is one of the critical activities that is strongly influenced by salinity stress, with its impacts varied based on elements including the plant species, the amount of salt, and other environmental conditions (Hnilickova et al., 2021). Several research has shown that adding NPs to plant leaves significantly increases the amount of chlorophyll present. According to studies, using manganese NPs (Mn-NPs) can help sustain a healthy rate of photosynthesis even when faced with severe abiotic stress conditions (Ye et al., 2020). In *Vigna radiata* plants under salt stress, manganese augmentation was found to improve several variables, including the membrane stability index, the amount of chlorophyll, and the activity of the enzyme nitrate reductase (Shahi and Srivastava, 2018). The exogenous treatment of Cu was observed to be valuable in reducing the negative effects of salinity on photosynthesis and water relations in maize plants (Iqbal et al., 2018). In *Brassica*, cerium NPs enhanced both biomass and photosynthetic efficiency when compared to plants that weren’t treated (Rossi et al., 2016). According to studies, Ag NPs to plants under salt stress can increase their chlorophyll levels and improve their fluorescence properties. Furthermore, Cao et al. (2017) showed that the incorporation of 100 mg/kg of CeO_2_ NPs in the growth medium accelerated photosynthesis and promoted plant development by controlling water usage efficiency, particularly in drought-stressed environments.

### 4.7 Strengthen antioxidant defense and remove ROS toxins

Insufficient water intake leads to the generation of ROS, H_2_O_2_, and MDA, which in turn triggers oxidative stress (Sutulienė et al., 2021). The remarkable ability of NPs to increase antioxidant activity helps to alleviate the negative consequences of water stress. The introduction of NPs (Si, ZnO, and Se) led to a substantial upsurge in the activity of APX, SOD, and CAT enzymes, which led to decreased oxidative harm triggered by shortage of water (Sun et al., 2020). The use of zinc oxide NPs has been found to improve the non-enzymatic actions of antioxidants like phenolic compounds and ascorbic acid (AsA). These compounds act in harmony with the antioxidant enzymes, including APX, CAT and SOD (El-Zohri et al., 2021). The foliar spray of SiO_2_ NPs at concentration 0, 12.5 ppm, 25 ppm, and 50 ppm considerably boosts non-enzymatic activities in plants under drought stress by increasing ferric reducing antioxidant power total phenolic content, 2,2-diphenyl-1-picrylhydrazyl scavenging activity, and total phenolic content, 2,2-diphenyl-1-picrylhydrazyl scavenging activity, ferric reducing antioxidant power (Sutulienė et al., 2021). Plants are protected from oxidative stress by NPs, which cause the buildup of antioxidant genes, osmolytes, minerals, and amino acids (Mittal et al., 2020).

Certain NPs exhibit characteristics comparable to some antioxidant enzymes, enabling them to help plants combat oxidative conditions. For instance, cesium, Mn, Cu, and Fe NPs exhibited POD-like capabilities, while cobalt, iron, and cesium NPs show CAT-like properties (Rico et al., 2015). Their investigation suggested that exposure to these NPs significantly improved the plant’s growth features. This improvement was associated with an upsurge in the activity of antioxidant enzymes, such as SOD, CAT and GPX as well as a reduction in the K^+^/Na^+^ ratio. Additionally, it was shown that using Ce-NPs augmented the activity of antioxidant enzymes in cotton plants, assisting in the elimination of ROS from within the cells. Additionally, the NPs supported plant development despite saline stress (Liu et al., 2021). A recent study examined the impact of applying iron nanoparticles (Fe-NPs) to *Dracocephalum moldavica* L. and found that it has the potential to enhance plant growth under salt stress conditions. The study showed that Fe-NPs increased the levels of flavonoids and phenolic compounds in the roots, which in turn contributed to improved plant development. In addition, it was shown that the NPs decreased the activity of antioxidant enzymes (Moradbeygi et al., 2020).

### 4.8 Enhance stress-responsive gene expression

NPs treatment causes a considerable upregulation of genes that respond to drought, including GmRD20A, GmDREB2, GmMYB118, and GmMYB174 (Kandhol et al., 2022). According to Yang et al. (2018), applying ZnO-NPs and CuO-NPs considerably intensified the expression of genes associated with drought resilience of wheat plant roots. Additionally, *Catharanthus roseus* plants cultivated under drought stress circumstances improved their antioxidant capability and activation of genes linked to alkaloid production as a consequence of the administration of CS-NPs (Ali et al., 2021a). Additionally, using ceria-based NPs caused kidney beans to express more proteins linked with stress resistance while simultaneously downregulating the proteins in charge of nutrition storage and glucose metabolism (Majumdar et al., 2015). GmWRKY27, 118, and 174 was also showed increased expression by NPs, which promoted hormone signaling, the formation of seed germination, lignin, and secondary metabolites in response to shortage of water (Rushton et al., 2010). The enhanced GmWRKY27 gene expression in plants subjected to NPs suggests a link between NPs and the control of ABA production and stomatal function under drought stress circumstances (Linh et al., 2020). The utilization of NPs resulted in a widespread elevation of the expression of genes linked to the ability to withstand water stress. The higher concentrations of copper (Cu) and zinc (Zn) were connected with the increased expression of genes correlated with metal stress in the shoots of plants treated with ZnO or CuO NPs. This finding implies that plants exposed to CuO or ZnO NPs defended against various threats, including drought (Khan et al., 2019).

The foliar treatment of Zn-NPs at 0, 20 and 80 mgL^−10^ to the leaves of rapeseed plants under salt stress produced alterations in the expression of genes associated to stress response such as SKRD2, MYC, and MPK4 showed decreased expression, whereas ARP and MPK showed increased expression (Hezaveh et al., 2019). These genes influence a range of hormonal, developmental, and physiologic reactions. Notably, the transcription factor-related genes MYC and SKRD2 showed higher expression and supported improved tolerance to abiotic stress (Hezaveh et al., 2019). Silicon-NPs have positively impacted hemp under salt stress, stimulating improved development and causing molecular alterations in this plant species (Guerriero et al., 2021). Silicon NPs were found to affect genes associated with cytochrome b6f (Cytb6f), ATP-synthase complex and the light-harvesting complexes in tomato plants subjected to salt stress, as revealed by a proteomics study. The treatment of silicon NPS changed the expression of 29 genes, kinase/phosphatase genes comprising transcription factors, genes involved in photosynthetic processes, and genes associated with stress. The impact of silicon also extends to the control of genes involved in manufacturing nitric oxide and auxin (Tripathi et al., 2021a). Furthermore, silicon NPs reduce the impacts of salt stress by varying the expression of genes like OsNCED, and OsZEP, which are essential for manufacturing the hormone ABA. These genes include OsZEP, OsNCED, and OsZEP (Tripathi et al., 2021b). Under salinity stress, silicon NPs have been discovered to promote salt transport into the vacuole by upregulating the protein OsHMA3, which improves plant development. Furthermore, silicon shields plants from stress by boosting activity of antioxidant enzymes (Siddiqui et al., 2020). The downregulation of salt stress genes (DDF2, MAPK3 and RBOH1) upon exposure to S NPs led to an improvement in the salt resilience of tomato plants (Almutairi, 2016a).

## 5 Nanoparticles and genetic engineering

The cutting-edge development in plant science, known as plant genetic engineering, is a key instrument to increase crop quality and production, boosting secondary metabolite levels in medicinal plants, and growing sustainable crops. Commonly, *Agrobacterium* and gene gun methods are used to transfer the gene of interest, although plant cell walls resist entering nucleus. Agrobacterium exhibited host specificity while gene gun method has potential to damage the plant tissues. In recent days, carbon nanotubes, magneto faction, DNA nanostructure, clay nanosheets, peptide NPs, CRISPER Cas9 and *de novo* transgenic plant production have accelerated progress in crop improvement (Lv et al., 2020). The usage of magnetic NPs has primarily been seen in medical research and animal science (Nasiri et al., 2020). However, research also exhibited the effective application of magnetoreception in plants (Zhao et al., 2017) established a quick and simple process utilizing magnetic NPs to produce transgenic seeds without needing tissue culture regeneration.

Additionally, SWCNTs exhibit effective membrane penetration via organelle walls (Hendler-Neumark and Bisker, 2019). The genetic transformation of chloroplasts and mitochondria efficiently gets around the restriction of gene migration, subsequent in increased herbicide tolerance in weeds because of their maternally transmitted nature (Lv et al., 2020). Kwak et al. (2019) developed SWCNTs complexed with chitosan specifically designed for the targeted delivery of DNA to the chloroplasts of numerous plant species, including spinach tobacco, watercress, and arugula. Similarly, Zhang et al. (2019) presented DNA NPs as a ground-breaking stage for siRNA delivery in plants. The researchers used the intensity of a fluorophore to gauge the internalization of these nanostructures into plant cells. This fluorophore was joined to the DNA strands at the connection sites of the DNA nanostructures through hybridization, enabling detection and analysis (Lv et al., 2020). NPs have great significance as materials for delivering biomolecules into cells due to their ability to cross biological membranes, protect and release a variety of payloads, and achieve varied targeting through chemical and physical changes. The first demonstration of the co-delivery of DNA and chemicals to *Nicotiana tabacum* plants in 2007 was done by Torney et al. employing biolistic delivery of gold-capped mesoporous silica NPs (MSNs) that vary in size from 100 to 200 nm (Torney et al., 2007). Mesoporous silica NPs (MSN), which were capped with gold NPs by covalent bonding, had a chemical expression inducer inserted into their pores (3 nm) (Cunningham et al., 2018). Additionally, recent studies have demonstrated the use of carbon nanotubes (CNTs) for the direct transport of plasmid DNA and siRNA into a variety of plant species, including model and non-model plants (Demirer et al., 2019). NPs have begun to play a crucial role in enabling and enhancing genome editing procedures by permitting effective and accurate transport of plasmids, RNA, and ribonucleoproteins (RNPs).

### 5.1 CRISPERCas system

Genome editing is gaining popularity for precise modifications of specific sequences and studying biological processes and plant genetics. CRISPER Cas, ZFNs and TALENs are three essential components used to insert specific genetic mutations into plants in crop improvement (Doman et al., 2020). In contrast, CRISPER Cas technology is a faster and easier genome editing method which suggests various horizons for crop improvement against abiotic stresses (Gao et al., 2020). Sg-RNA initiate Cas9 nuclease to form complex of Cas9-sgRNA at targeted genomic DNA and spark the cleavage. Hence leads to the development of double stranded breaks with the help Cas9 nuclease in DNA of plant (Shan et al., 2013). The successful stories of CRISPER Cas9 technology were revealed by three research groups on rice, wheat, tobacco and Arabidopsis (Nekrasov et al., 2013). Afterwords, this technology immensely used in various project and objectives like powdery mildews in wheat to regulate TaMLO homologs (Wang et al., 2014). Similarly in maize, yield was improved by editing waxy alleles by CRISPERCas9 successfully (Gao et al., 2020). The utilization of CRISPR-Cas system has opened new avenues for crop breeding, genetics, and genomics. Its precise and efficient genome editing capabilities have enabled researchers to make targeted modifications in the genome of crops, leading to the development of improved crop varieties with desirable traits. This technology has revolutionized the field of crop improvement and holds great promise for the future of agriculture. This technology has the potential to improve specific traits in plants while minimizing the risk of unintended effects, making it useful in addressing both biotic and abiotic stress factors.

## 6 The toxicity of nanoparticles in plants and biological environment

**Figure 5:**
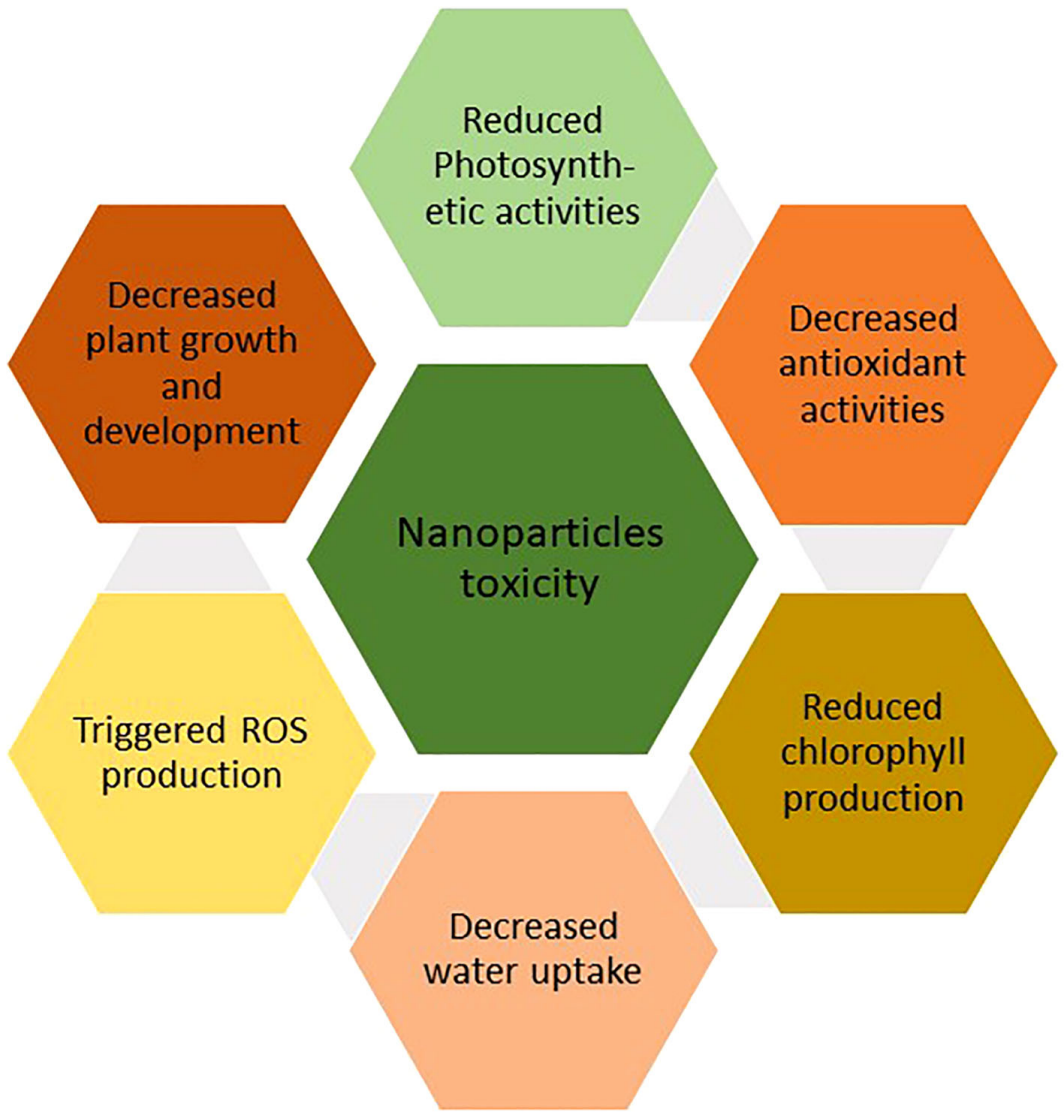
Impact of NPs toxicity on plant growth.

ROS are generated due to interactions of NPs with plants through numerous physical and chemical processes (**Figure 5**). Increased ROS generation carried by NPs can negatively impact plant cells, severely limiting plant growth and development (Xie et al., 2019). The shape, size, and characteristics of the NPs regulate their hazardous effects. Metal oxide NPs like Fe_3_O_4_, ZnO, and TiO_2_, which have applications in a variety of sectors, as well as metal NPs like gold, platinum, silver, and iron, have the potential to endanger human health. These NPs can harm proteins, DNA, and cell membranes when they come into contact with cells, significantly reducing plant development (Hsin et al., 2008).

### 6.1 Nanoparticles inhibit plant growth

Soil is a significant source of NPs, and plants use active transport systems in their roots to take up both NPs and nutrients from the soil (Khan et al., 2021). NPs are uptake by the roots and pass through the root cortex and epidermis of cell walls before reaching the upper parts of the plant and causing adverse effects inside the plant (Rajput et al., 2018). NPs might negatively affect plant development at higher concentrations by diminishing antioxidant activities, decreasing photosynthetic effectiveness, and decreasing chlorophyll production. NPs have the benefit of being substantially smaller than normal bulky materials, enabling them to be immersed into biological systems near 15-20 times more quickly (Khan et al., 2021). Research suggests that the toxicity of NPs in various microorganisms such as plants and algae is linked to physical damage and the production of ROS, leading to oxidative stress (Hou et al., 2018). The colour structure of photosynthesis, the effectiveness of PS-II (photosystem II), and the development of aquatic plants like amphibian plants can all be impacted by NPs in aquatic environments (Jacobasch et al., 2014).

The agriculture industry cannot fully embrace and implement nanotechnologies due to increasing concerns about the bioavailability and toxicity of NPs and the shortcomings of the current regulatory framework (Ali et al., 2021a). According to Rao and Shekhawat (2016) improper use of NPs can negatively impact plant development, such as reducing the production of protein and pigment in plants. Plant cells also develop extra substances like metallothioneins and phytochelatins due to the stress produced by NPs. These substances defend against the damaging impacts of oxidative stress on plant cells (Dev et al., 2018). NPs in the soil environment go through a sequence of bio/geo transformations that ultimately regulate their toxicity and bioavailability (Ali et al., 2021a). NPs of various compositions adversely influence the roots and shoot elongation of seedlings, which is mostly associated with the uptake of NPs into the roots. Silver NPs (AgNPs) have cytotoxic effects on some plants and reduce germination, transpiration, shoot and root length, and involved changes in gene expression, oxidative stress and cell death (Thuesombat et al., 2014). The concentration of the NPs influences the extent that growth is inhibited in mung bean and sorghum (Lee et al., 2012). Inhibitory and toxic effects of NPs are listed in **Table 2**.

**Table 2:** Toxic and inhibitory effects of nanoparticles on plants.

NPS	Plant	Negative impact	Reference
ZnO	*Glycine max*	Superoxides were produced, leaf biomass decreased, gene expression was changed, and root elongation, cell viability, and biomass were all suppressed.	(Hossain et al., 2016)
ZnO	*Zea mays*	Reduced mineral nutrient uptake, photosynthesis, and root activity were caused by enhance in superoxide anions and a decreased in superoxide dismutase activity.	(Wang et al., 2016)
ZnO	*Zea mays*	Showed negative impacts on the development of seedlings as well as seed germination.	(Fellmann and Eichert, 2017)
ZnO	*Medicago sativa*	Reduced the biomass of the roots by 80% as a result.	(Bandyopadhyay et al., 2015)
ZnO	*Vigna angularis*	Caused plant physiology to be disturbed, increased oxidative stress, and decreased amounts of photosynthetic pigment.	(Jahan et al., 2018)
Ag	*Lysopersicon esculentum*	Lower fruit production, photosynthesis, and CO2 assimilation as a result of reactive oxidative stress that was induced.	(Das et al., 2018)
Ag	*Allium cepa*	Root growth was suppressed	(Pittol et al., 2017)
Ag	*Allium cepa*	Significantly reduced root development, stimulated the mitotic index, triggered the production of ROS, and in higher quantities led to oxidative DNA damage.	(Cvjetko et al., 2017)
Ag	*Lupinus termis*	Decreased RFW and SFW, decreased overall chlorophyll content.	(Al-Huqail et al., 2018)
CuO	*Zea mays, Oryza sativa*	At a dosage of 2000 mg/L, maize and rice showed 95% and 97% inhibition in root length, respectively.	(Yang et al., 2015)
CuO	*Brassica rapa*	Sugar and pigment production during photosynthesis was decreased.	(Chung et al., 2019)
CuO	*Oryza sativa*	Reduced water uptake by aerial parts and roots, grain yield decreased	(Peng et al., 2017)
TiO_2_	*Oryza sativa*	Low water absorption was seen in the root and aerial sections, which significantly reduced grain output.	(Du et al., 2017)
TiO_2_	*Cannabls sativa*, var. capitata, *Avena sativa, Lactuca sativa, Allium cepa*	Root development was impeded in the cases of oat, corn, cabbage and lettuce, while soybean and cucumber germination was diminished.	(Andersen et al., 2016)
TiO_2_	*Zea mays*	Suppression of root and shoot development as well as prevention of germination.	(Fellmann and Eichert, 2017)
TiO_2_	*Oryza sativa*	Reduced biomass and changes to the antioxidant defence systems.	(Wu et al., 2017)
Al_2_O_3_	*Sinapis alba*	All concentrations had a negative impact on seed germination.	(Landa et al., 2016)
Al_2_O_3_	*Triticum aseivum*	The synthesis of anthocyanin and photosynthetic pigments decreased as the H2O2 level rose.	(Yanık and Vardar, 2015)
Au	*Hordeum vulgare*	Reduced, leaf and root lengths, fresh plant biomass and necrotic and yellow roots and leaves.	(Feichtmeier et al., 2015)
Au	*Nicotiana xanthi*	Caused biotoxicity and the development of necrotic lesions on leaves.	(Wang et al., 2023)

### 6.2 Oxidative damage of nanoparticles

The increasing antioxidant enzyme activity is an indirect indication of increased cellular ROS levels. The plants containing NPs can protect biological components from oxidative stress by using phenols and phenolic acids (Yasur and Rani, 2013). Wang et al. (2011) investigated the effects of Fe3O4 NPs on cushaw pumpkin and ryegrass plants. They investigated how these NPs affected the production of ROS, which affected membrane stability. According to other research, releasing copper ions or copper oxide NPs might cause oxidative stress (Nair and Chung, 2015).

Nevertheless, the solubilization of copper oxide NPs within plant cells leads to the subsequent release of copper ions. Consequently, a redox process involving both Cu^2+^ and Cu^+^ ions leads to oxidative damage (Shi et al., 2011). Lipid peroxidation was shown to be primarily caused by ROS, which is produced by the Fenton reaction due to the existence of polyvalent CuO NPs (Fubini et al., 2007). Panda et al. (2011) supervised a study on *Allium cepa* and examined the formation of certain ROS, including superoxide radicals (O2%-) and H_2_O_2_. They discovered that the presence of silver NPs caused these reactive species levels to rise, which caused an oxidative burst in the plant. According to Atha et al. (2012), copper oxide NPs were discovered to cause DNA harm and prevent growth in radishes, and annual ryegrass. The association between titanium oxide NPs and DNA and the underlying mechanism causing DNA damage in onion and tobacco have been shown through the use of atomic force microscope imaging (Ghosh et al., 2010). Mukherjee et al. (2014) examined the membrane damage produced by zinc oxide NPs in *Pisum sativum* and observed a comparatively higher level of lipid peroxidation in plants exposed to NPs evaluated to the control (non-treated) plants. The study also reported that *Pisum sativum* plants treated to zinc oxide NPs produced an excessive amount of hydrogen peroxide (H_2_O_2_).

Dimkpa et al. (2012) carried out research to evaluate the promising impacts of CuO and ZnO NPs. The study showed a rise in lipid peroxidation levels and reduced chlorophyll content, indicators of oxidative stress. Compared to the corresponding control groups, the incidence of copper oxide NPs in wheat induced a nearly four-fold increase in lipid peroxidation, whereas the existence of ZnO NPs resulted in a two-fold increase. It was shown that cerium oxide NPs had an adverse effect on *asparagus lettuce*, causing membrane degradation, lipid peroxidation, cell membrane impairment, and inhibiting root elongation (Cui et al., 2014). Marusenko et al. (2013) they stated a decrease in chlorophyll content in *A. thaliana* plants when subjected to Fe NPs. Faisal et al. (2013) examined that NiO nanoparticle treatment of tomato plants caused oxidative stress to be induced in their roots. Additionally, flow cytometry analysis found that the protoplasts had a greater level of oxidative stress production. Higher levels of enzymes like CAT and SOD were associated with increased reactive stress. Shaw and Hossain (2013) demonstrated that copper oxide NPs affected both root and shoot development as well as photosynthetic system in *Hordeum vulgare.*

### 6.3 The impacts of NPs on human health and the environment

NPs are being used at an exponential rate; however, it’s important to remember that there may be harmful and toxicological impacts on environment and human health. NPs have the potential to be released into the environment through several processes, including manufacture, use, recycling, or disposal. These NPs may linger in biological systems, soil, water, or the air (Roy et al., 2014). NPs can enter the body of a person or an animal by skin contact, oral ingestion, or inhalation through the respiratory system, then spread to other bodily compartments. The activation of pro-inflammatory cytokines and chemokines upon exposure to NPs was discovered to result in the recruitment of inflammatory cells, which affects the immune system’s homeostasis and can cause autoimmune, allergy, or malignant illnesses (Roy et al., 2014).

Additionally, breathing in or exposure to ultrafine particles has been linked to several respiratory, cardiovascular, and central nervous system problems (Joudeh and Linke, 2022). Similarly, NPs can penetrate plant cells and have negative effects (Stark, 2011). For example, zinc oxide (ZnO) and aluminum (Al) NPs have been discovered to hinder the development of plant roots (Lin and Xing, 2007). Numerous NPs have been studied for their potential to cause immunological and cellular damage. The effect of NPs on human health has been examined using *in vitro* toxicity assessment models, such as cell cultures that include cancer cell lines. However, a substantial body of research on the impacts of nanomaterials on human health and the environment reveals that metal NPs can cause cytotoxic effects, which are determined by their charge (Pande and Arora, 2019). NPs can increase their toxicity at the nanoscale level by interacting electrostatically with biological membranes and various cellular metabolites in the cytoplasm (Prasad et al., 2017). According to research, when exposed to light and molecular oxygen, certain photochemically active NPs, including fullerenes, ZnO, SiO2, and TiO2, can directly transfer electrons to produce reactive oxygen species such as superoxide radicals. The result might be oxidative stress (Nayan et al., 2016).

## 7 Conclusion and future thrusts

Currently, the use of NPs is constantly growing and becoming indispensable in many industries, including agriculture. For sustainable agriculture, we need ecologically acceptable solutions to crop yield decline caused by drought and salt stress. Intensive research is underway to explore the possible role of various NPs in mitigating damage caused by drought and salinity stress to enhance plant development and crop yield. Due to their tiny size, NPs may easily enter plant tissues and have a favorable effect on morphology, physiology, and biochemistry of plant, promoting plant growth and increasing agricultural output, especially under drought and salinity stress situations. Moreover, the application of NPs greatly increases plant functioning and offers tremendous resilience to plants, enabling them to tolerate drought and salt stress efficiently. The application of NPs enhances membrane integrity, nutrient absorption, and defends the plant’s photosynthetic system from injury produced by salt and drought stress, subsequent augmenting plant development in these challenging circumstances. The utilization of NPs also enhances the production of phenolic compounds and hormones that protect plants from stress. The expression of antioxidant and stress-responsive genes is also increased by NPs, which considerably strengthens the defense mechanism against salinity and drought stress. The function of NPs in aiding numerous processes to promote resilience to drought and salt stress has recently been the subject of substantial investigation. The use of NPs in crop development and sustainable agriculture is still nascent, and the existing research in this field lacks consistency and is insufficient. The impact of NPs on seed germination remains unexplored, highlighting the need to investigate their role in germination mechanisms, encompassing water uptake, radical protrusion, and activation of enzymes responsible for food mobilization. Furthermore, it is critical to investigate how NPs affect their metabolic functions because gibberellins and abscisic acids are essential for seed germination. NPs enhance nutrient absorption in plants under drought and salinity stress, but further research is required to fully understand their function in nutrient channels and ionic transporters in plants under such stresses. The treatment of NPs substantially protects the photosynthetic apparatus, but further research is needed to determine how they affect stomata motions, anion channel control, and intercellular signaling in guard cells of plants under salinity and drought stress. The investigations on the effects of NPs on proteome may be advantageous to understand better the numerous processes by which NPs promote drought and salinity tolerance. The impacts of NPs on genetic and proteomic parameters have not received enough attention, underlining the need to investigate these topics in future studies. The synergistic potential of combining microorganisms with NPs to promote drought and salinity tolerance also constitutes an interesting research direction. Additionally, understanding the interactions between NPs and plants would help us understand how plants cope with drought and salt. The timing and content of NPs under diverse climatic circumstances must be optimized to meet the unique needs of distinct crops. While the bulk of research focuses on the impacts of NPs on plants under salt and drought stress, it is critical to examine the influence of NPs on plants exposed to various stressors at altered times throughout their life cycle.

## Author contributions

AR: Data curation, Writing – original draft. SK: Writing – original draft. FS: Data curation, Writing – review & editing. ZPe: Conceptualization, Data curation, Visualization, Writing – review & editing. KF: Writing – review & editing. NW: Writing – review & editing. YJ: Conceptualization, Writing – review & editing. ZPa: Conceptualization, Writing – review & editing. SH: Data curation, Writing – review & editing. LW: Data curation, Writing – review & editing. AQ: Conceptualization, Data curation, Writing – review & editing. XD: Conceptualization, Supervision, Writing – review & editing. HL: Supervision, Writing – review & editing.
